# Page Turning Using Assistive Robot with Low-Degree-of-Freedom Hand

**DOI:** 10.3390/s24196162

**Published:** 2024-09-24

**Authors:** Hidetoshi Ikeda, Yuta Mizukami, Masahiro Sakamoto, Takumi Saeki, Hokyoo Lee, Masakazu Hori

**Affiliations:** 1 Faculty and Department of Engineering (Mechanical and System Engineering Field, Advanced Manufacturing Robotics and System Control Course), Niigata Institute of Technology, Kashiwazaki City 945-1103, Japan; 2 National Institute of Technology, Toyama College, Toyama 939-8045, Japan

**Keywords:** page-turning, low-degree-of-freedom robotic hand, hand mechanism, foldable robotic hand, assistive robot

## Abstract

This paper proposes a page-turning strategy using an assistive robot that has a low-degree-of-freedom robotic hand. The robotic hand is based on human object handling characteristics, which significantly reduces the number of fingers and joints required to handle various objects. The robotic hand has right and left planar fingers that can transform their shape to handle various objects. To turn a page, the robot uses the planar fingers to push the surface of the page and then rotates the fingers. The design concept, mechanism, sensor system, strategy for page turning, and control system of the robotic hand are presented. The experimental results show that the robot can turn pages using the proposed method; however, it sometimes failed to turn the page when the robotic hand height was too low and too close to the book because the rotation of the fingers was stopped by the book. When the hand detects excessive force during page turning, the control system changes the shape of the fingers and releases the force from the book. The experimental results show the effectiveness of the control system.

## 1. Introduction

Many assistive robots have one or more manipulators with a hand mechanism. Multi-finger robotic hands, which have been extensively studied [1], can handle objects by appropriately extending or bending the fingers. Such hands have been installed on assistive robots. Various types of multi-finger hands have also been developed for industrial use. Mouri et al. developed an anthropomorphic robotic hand, meaning a human-like robotic hand [2]. Many other multi-finger hands have been developed, and their effectiveness has been demonstrated. Robotic hands with two fingers and two axes [3], two fingers and five axes [4], and four fingers [5] have been developed. Many studies have been conducted on the use of anthropomorphic robotic hands as prostheses [6,7,8].

Multi-finger robotic hands have the potential to perform advanced tasks. However, their structure is complex, and thus they are difficult to control. In the design of such hands, the numbers of fingers and joints are thus often minimized without affecting the handling capabilities. Numerous under-actuated robotic hands or grippers that have few actuators for driving joints have been developed [9,10,11,12,13].

Because the inside of a finger is narrow, tendon-driven robotic hands have been widely researched [14]. Tendon-driven mechanisms include an integrated linkage-spring-tendon-compliant anthropomorphic robotic hand [15,16]. A robotic hand with under-actuated robotic fingers that can open a bottle cap and pour coffee has been developed [17].

Robotic hands with soft fingers have also been developed. Soft hands can handle delicate objects without damaging them [18]. Soft hands with four soft fingers for handling groceries [19] and soft robotic hands that can sort fruit have been studied [20,21]. Soft hands or grippers for handling marine life [22,23] and soft robotic arms for use as prosthetic hands [24] have also been developed.

A robotic hand that uses the jamming phenomenon of a granular material instead of multiple fingers has been studied [25]. A robotic hand that uses both a jamming transition and a tendon drive has also been investigated [26].

A gripper mechanism that consists of many pins, allowing it to conform to the shape of any object, has been developed [27].

Multi-finger hands with additional functions that human hands lack have been studied. Research has been conducted on a hand that has two fingers and a sliding mechanism for precise handling using the surface of the fingers [28], a hand with a mechanism that allows four fingers to fully rotate [29], and a hand with a ball mechanism at the tip of each finger [30].

A lot of effort has also been devoted to the development of robotic hands. Several reviews, such as that by Bicchi [31], summarize research on various types of robotic hands.

Humans perform various tasks by bending their fingers and changing the shape of their hands. Many studies have classified the grasping mechanism of the human hand and its capabilities [32,33,34,35,36].

The present authors previously designed a robotic hand that has two planar fingers with joints that have a wide movement range. The hand has six degrees of freedom (DoFs), allowing it to perform various tasks. Strategies for pinching, grasping an object [37], and retrieving a file binder using the robotic hand were presented [38]. Expanding the applicability of low-DoF hands will allow for the simplification of robotic hand systems. The present study proposes a strategy for page turning using a hand mechanism.

Many studies have been conducted on page turning. Ueda et al. proposed a page-turning method that uses a robotic hand with two fingers, each of which has two DoFs [39]. The robot pinches a page to turn it. A page-turning machine for disabled people has been developed [40]. Clips are placed on each page, and an electromagnet mounted on a robotic arm lifts and turns pages. A mechanism for turning pages using cross-shaped links has been developed [41]. A page turner that lifts a page and then blows air to turn it has also been developed [42].

A wearable page turner that uses a rod has been designed for people with upper limb disabilities [43]. Mochizuki proposed a page-turning mechanism that uses a friction roller for automated teller machines [44].

## 2. Materials and Methods

### 2.1. Design Concept for a Foldable Robotic Hand

**Figure 1:**
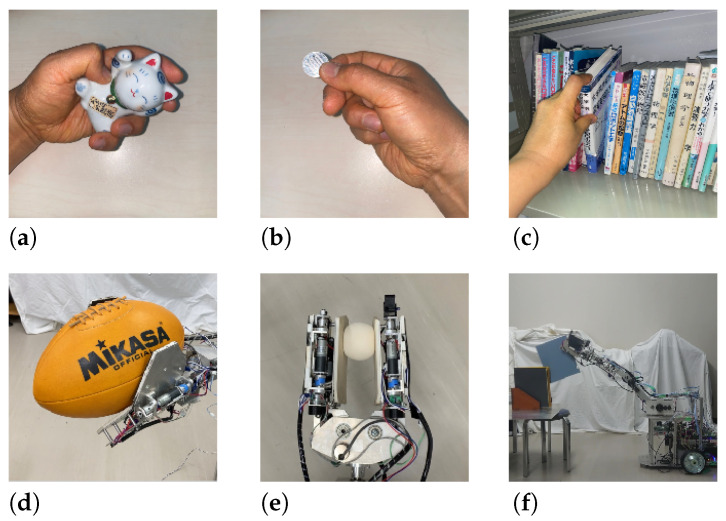
Object handling by human hand and concept of robotic hand. (**a**) Grasping of object by human hand, (**b**) pinching of object by human fingers, (**c**) tilting of book by human fingers, (**d**) grasping of object by robotic hand, (**e**) pinching of object by robotic hand, and (**f**) retrieval of file binder from book shelf by robotic hand.

Humans change the shape of their hands by bending and stretching their fingers to control various objects. For example, when grasping (Figure 1b), the hand conforms to the shape of the object to maintain a wide contact area. A wide contact area is also maintained when an object is held in the palm of the hand. The action of holding an object in the palm of the hand may be less robust to disturbances than the action of grasping. Nevertheless, it is possible to handle and move an object in the palm if it is relatively small compared to the hand. One or two fingers can be used to exert a force on an object at a few points to pinch (Figure 1b), claw (Figure 1c), or roll the object. These commonly used actions are sometimes unreliable.

In the case of a force acting on an object within a plane, if the state of grasping is not changed frequently, such as in the case when grasping an object, lifting it up, and placing it in another location, there is no need for continuous finger movement. A finger is moved only to conform to the shape of the object. A simple hand mechanism that has simple planar fingers can perform such a task.

In the case of handling an object using one or more fingertips, the remaining fingers do not exert a force on the object.

**Figure 2:**
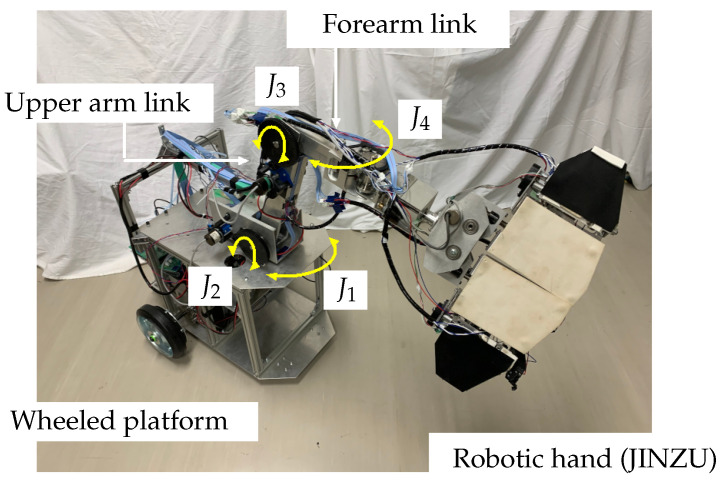
Photograph of whole robot.

Taking into account these human handling characteristics, the authors previously devised a robotic hand concept that can reduce the number of DoFs of fingers by properly setting the multi-finger hand’s shape, arrangement, and range of joint motion. The authors previously developed the robotic hand JINZU, which has six DoFs (Figure 1d–f). JINZU has left and right planar fingers, which have a wide range of joint movement. The hand mechanism is attached to manipulator links, and the manipulator is installed on a wheeled platform (Figure 2).

This robotic hand can manipulate various objects by applying force within a plane (Figure 1d), at a few points (Figure 1e), or at one point (Figure 1f) by changing its shape. To accomplish all of these tasks, a robot hand with many degrees of freedom is generally required. However, the robotic hand that we developed can accomplish such tasks with only six degrees of freedom by using right and left planar fingers that have a wide range of joint movement, allowing the shape of the hand to be transformed. Such a concept has not been previously proposed. The ability of this hand to turn the pages of a booklet is reported herein.

### 2.2. Mechanism of Robot and System Configuration

The robot was developed in our laboratory (Figure 2). It has a wheeled platform. The manipulator, which is attached to the platform, consists of an upper link, a forearm link, and a hand. The platform has two pairs of wheels, each of which consists of a left wheel and a right wheel. The two drive wheels at the rear are driven individually; the front wheels are casters. Two motors (Tsukasa Electric Co., Ltd., Tokyo, Japan, TG-85E-SU-47.9-KA, 24 V, Nakano-ku) and two encoders (Autonics, Haeundae-gu Busan, South Korea, E30S4-100-3-N-5) are used in the driving system.

**Figure 3:**
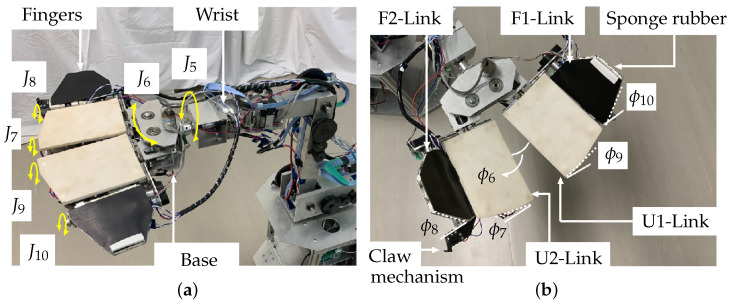
Robotic hand used in this study. (**a**) Three parts of hand mechanism and (**b**) right and left fingers.

The manipulator has two shoulder axes, $J_{1}$ and $J_{2}$, and two elbow axes, $J_{3}$ and $J_{4}$ (Figure 2). Motors and encoders are installed on each axis (for $J_{1}$, $J_{2}$, and $J_{3}$, the motor is Tsukasa Electric Co., Ltd., TG-85E-KU-113-KA, 24 V, and the encoder is Nidec Copal Electronics, RE12D-100-201-1; for $J_{4}$, the motor is TG-101C-GU-581-KA, 24 V, and the encoder is RECW20D-25-201-1). The robotic hand consists of three parts, namely, the fingers, finger base, and wrist (Figure 3a). The wrist has an axis, $J_{5}$, that can rotate the whole mechanism of the finger base and fingers. A motor and an encoder (motor: Tsukasa Electric Co., Ltd., TG-101C-GU-581-KA, 24 V; encoder: Nidec Copal Electronics, RE30E-360-213-1) are attached to the wrist.

The base has two finger joints that are controlled by a motor and an encoder (motor: Tsukasa Electric Co., Ltd., TG-101C-GU-581-KA, 24 V; encoder: Nidec Copal Electronics, Shinjyuku-ku, Tokyo, Japan, RE12A-100-100-1) (Figure 3b). The base can change the finger angle. The hand has right and left fingers, whose shape is planar. Both fingers are attached to the finger base. Each finger has two links, namely an upper link (U-Link) and a forearm link (F-Link). The left and right forearm links are denoted F1-Link and F2-Link (Table 1), respectively, and the left and right upper links are denoted U1-Link and U2-Link, respectively. Motors and encoders are installed in the U-Links to control the fingers ($J_{7}$–$J_{10}$; motor: Tsukasa Electric Co., Ltd., TG-85E-KU-113-KA, 24 V; encoder: Nidec Copal Electronics, RE12D-100-201-1). The joint angles of the U-Links ($\phi_{7}$ and $\phi_{9}$) are −90° $\leq \phi_{7}\leq 90$° and −90° $\phi \leq \phi_{9}\leq 90$°, respectively, and those of the F-Links ($\phi_{8}$ and $\phi_{10}$) are −180° $\leq \phi_{8}\leq 60$° and −180° $\leq \phi_{10}\leq 60$°, respectively. A claw mechanism, which was made using a 3D printer and is used to snag, scrape, or tilt an object, is attached to the side of the hand. When the claw makes contact with an object, its link is bent, pressing a microswitch (Omron Corp, Kyoto, Japan, D2MQ-01L-D, D2MQ-4L-1) that detects the contact.

**Figure 4:**
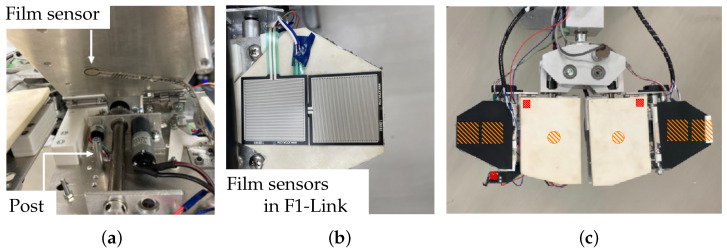
Sensors installed in the U-Links and F-Links. (**a**) Force (film) sensor inside a U-Link, (**b**) force (film) sensor inside an F-Link, and (**c**) positions of microswitches (small red squares) and force sensors in finger (orange circles and squares).

**Figure A1:**
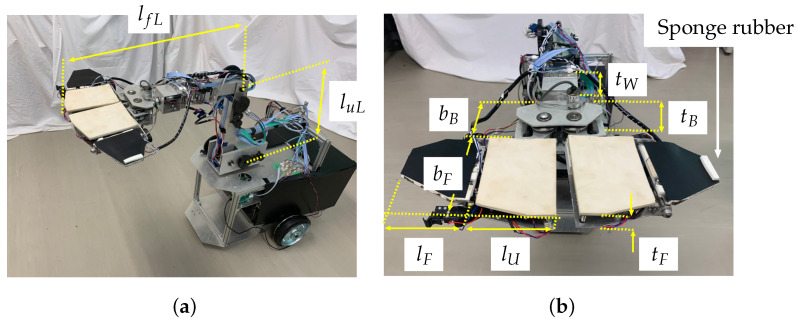
Definitions of manipulator and hand mechanism size parameters. (**a**) Manipulator links. (**b**) Hand mechanism components.

Sponge rubber parts (Misumi Corp, Chiyoda-ku, Tokyo, PRGCW5, white) are glued on the front sides of the U-Links and the F-Links, and rubber parts (2 mm thick, black) are glued on the sponge rubber parts of the F-Links (Figure 3b). Force sensors (film sensors, Tekscan Corp., Boston, MA, USA, A201, High 445 N, 0–100 lb [0–45.36 kg]) are attached inside the U-Links (Figure 4a). When the U-Links make contact with an object, the posts push the force sensors, allowing the robot to detect the contact. Four force sensors (film sensors, Interlink Electronics Inc. CA, United States, FSR406, Figure 4b) are installed inside the front of each F-Link. The sensors are attached to the sponge rubber parts (white) and covered by the rubber parts (black). In addition, sponge rubber (white) cut into strips is attached to the tip of the F1-Link (length: 50 mm, width: 10 mm, thickness: 5 mm; see Figure A1) to increase force sensor responsiveness and thus facilitate page turning (see Section 3).

Three microswitches (Omron Corp., D2F-01L-D) are installed on the inside of the fingers and claw mechanism. The force sensors and microswitches detect contact with an object. Figure 4c shows the position of the film sensors and microswitches. The orange squares and circles show the positions of the film sensors described above (Figure 4a,b). The positions of microswitches are shown by small red squares.

**Figure 5:**
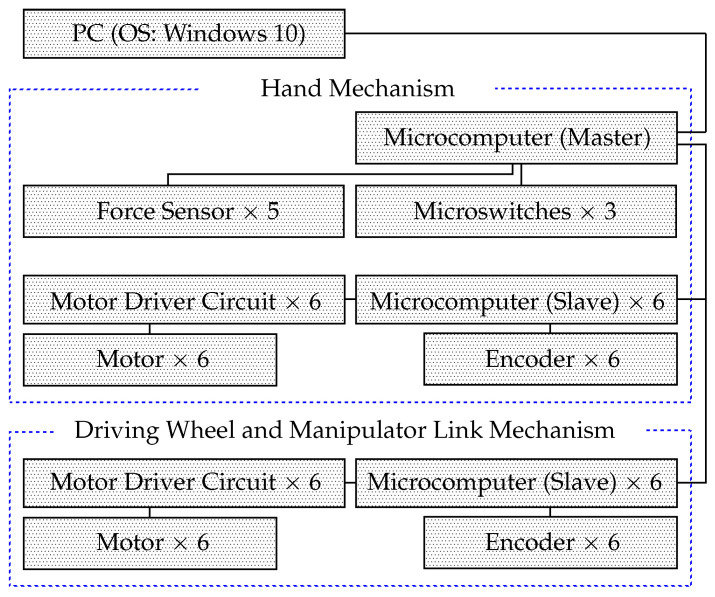
Configuration of the robot control system.

Figure 5 shows the system configuration of the robot. The two motors and encoders are attached to individually driven wheels. They are installed on the shoulder and elbow axes of the manipulator ($J_{1}$–$J_{4}$, Figure 2). A motor is connected to each of the six motor driver circuits (Cytron Co., Ltd., Pulau Pinang, Malaysia, MD10C), each of which is connected to a microcomputer (Arduino Holdings, Arduino Leonardo, Torino, Italy). An encoder is connected to each microcomputer. The microswitches and force sensors (film sensors, Figure 4a,b) are connected to the master microcomputer. Twelve microcomputers are used as slave computers. Five force sensors, three microswitches, and an ultrasonic sensor on the hand are connected to another master microcomputer. The master microcomputer is connected to a PC running Windows 10. The software running on the microcomputers (Arduino Leonardo) was developed in the Arduino language.

## 3. Results and Discussions

### Strategy for Page Turning

**Figure 6:**
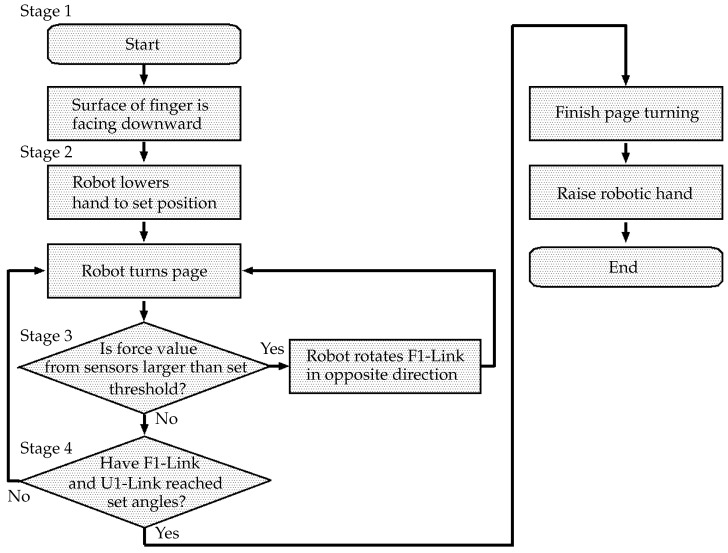
Flowchart of page turning.

A booklet was placed in front of the robot. It was assumed that the robot knew the position of the booklet. The booklet was a catalog of mechanical parts (length: 257 mm, width: 182 mm, thickness: 46 mm). The page thickness was 0.025 mm, and the coefficient of friction was 0.62. Figure 6 shows a flowchart of the process of turning a page, which is divided into four stages (1–4). It is assumed that the coefficient of friction between the paper and the robot hand is sufficiently high for slippage not to occur.

**Figure 7:**
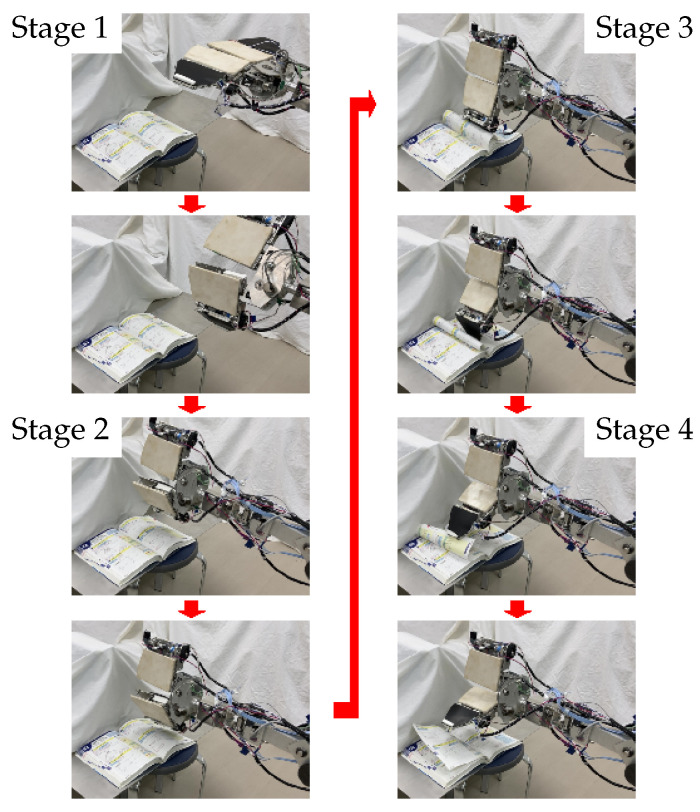
Snapshots of page-turning experiment.

In Stage 1, the robot prepares to turn the page. In Stage 2, the robot starts to turn the page. In Stage 3, the hand turns the page, with the finger angle controlled according to the height of the booklet. In Stage 4, the end of page turning is recognized, and the process is stopped. The details of the page-turning process are described below. Snapshots of the process are shown in Figure 7.

**Figure 8:**
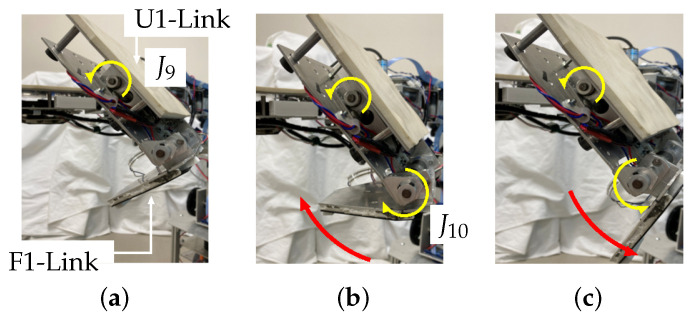
Control of finger. (**a**) $J_{9}$ rotates left finger to turn page. (**b**) The F1-Link is rotated in the opposite direction ($J_{10}$) when the film sensor detects a force value larger than the set threshold. (**c**) When the force exerted by the book becomes smaller than the set threshold, the rotation direction of the F1-Link returns to the page-turning direction.

**Stage 1**  The process of page turning starts. The robot drives the wrist joint, $J_{5}$, and faces the surface of the F1-Link downward. If necessary, the robot moves forward.
**Stage 2**  The robot lowers the hand above the booklet. It drives the $J_{9}$ axis and turns the page using the U1-Link and the F1-Link. When the hand touches a page, the film sensor of the F1-Link detects it.
**Stage 3**  First, the robot rotates the U1-Link in the direction required to turn the page ($J_{9}$, Figure 8a). When the surface of the hand comes into contact with the book, the film sensor of the F1-Link (Figure 4) detects the force exerted by the page. Here, if the height of the finger is too low, the finger will collide with the book (even if the robot keeps rotating the finger around $J_{9}$), and the trajectory of rotation will be limited; thus, page turning will fail. Therefore, if the film sensor detects a force larger than the set threshold, the U1-Link continues to rotate in the direction to turn the page, but the F1-Link rotates in the direction opposite to that of page turning ($J_{10}$, Figure 8b). If the sensor then detects a force smaller than the set threshold, the F1-Link returns to rotating in the direction of page turning (Figure 8c). In this way, the force detected by the film sensor on the F1-Link is used to avoid stopping the rotational motion of the hand caused by contact during page-turning.
**Stage 4**  The finger continues to rotate and turn the page. When the F1-Link and the U1-Link rotate to the set position, the page-turning process ends, and the robot raises the hand.

A page-turning experiment was conducted using the robotic hand (see Video S1). A book was placed on a chair with a height of 400 mm. It was assumed that the robot knew the height and horizontal position of the book. Page turning was performed using the process described above.

In Stage 1, the robot moved forward in the direction of the book and faced the finger surfaces downward.

In Stage 2, the robot lowered the robotic hand to a predetermined height (200 mm from the book to the center of the two fingers) and rotated the U1-Link and the F1-Link for page turning and turned a page. The experiment showed that the robot could turn a page. However, after several trials, it was found that, when the height at which the hand was lowered was too low, the rotation trajectory around $J_{9}$ (axis of the upper link of the right finger) of the fingers was interfered with by the booklet, and page turning could not be accomplished.

Therefore, sponge rubber (white, the same as that attached to the U-Links) cut into strips was attached to increase the responsiveness of the film sensors installed on the F1-Link and to eliminate hand height errors.

Furthermore, recall that when the force exerted by the book detected by the film sensors in the F1-Link is larger than the set threshold, the F1-Link rotates in the direction opposite to that of page turning. A control process that performs page turning by decreasing the radius around $J_{9}$ and traces the surface of the book with the fingers (Stage 3) was added. This enabled stable page turning. In the process, when the force detected by the film sensor is smaller than the set threshold, the rotation direction of the F1-Link returns to the page-turning direction.

In Stage 4, the robot recognized the end of the page turning when the positions of the U1-Link and the F1-Link reached their set values. The robot then raised the hand and ended the operation.

## 4. Conclusions

This study proposed a method for page turning using a robotic hand. Even though the hand has only six DoFs, it can perform various handling actions, such as pinching, grasping, and clawing.

A page-turning experiment conducted using the robot confirmed that the proposed method is effective. However, it was found that, when the height of the robotic hand at the start position of page turning was too low, the rotational trajectory of the fingers was limited by the book, making it impossible to turn the page. Therefore, sponge rubber was added on the tip of the finger. Furthermore, a system to control the fingertip link to release the force from the book by deforming the shape of the hand, which involves stroking the page surface and turning the page, was developed. The performance of the system was evaluated in an experiment involving turning the pages of a catalog of mechanical parts whose page thickness was 0.025 mm and whose coefficient of friction was 0.62. The results of multiple trials showed that the proposed device could successfully complete the page-turning task.

As described above, the feasibility of the proposed page-turning method was confirmed. Nevertheless, there is much room for improvement to realize highly accurate page turning, such as clarifying the applicability of various qualities to paper, improving adaptability, and adding the ability to recognize book layouts. This study showed that page turning can be performed by deforming a low-DoF hand mechanism.

In the future, the applicability of various qualities to paper and the relationship between the optimal force applied to the page by the hand and the friction coefficient will be clarified. In addition, we are planning to analyze the kinematics, statics, and dynamics of the system and report on the hardness, material properties, and dynamics of various types of paper, in addition to performing related experiments. We will also analyze the dynamic changes of the robotic hand and the trajectory while page turning. In addition, a control system will be developed to handle cases in which slippage of the paper occurs while turning the page.

A system that can recognize the book layout will also be developed. The results will be used to construct an accurate page-turning system. Finally, theoretical analyses and experiments will be conducted to determine the limitations of the robot’s performance and ways to improve it.

## Figures and Tables

**Table 1:** Abbreviations (see Figure 3).

F1-Link	Right forearm link of finger
F2-Link	Left forearm link of finger
U1-Link	Right upper link of finger
U2-Link	Left upper link of finger

## Data Availability

The data presented in this study are available on request from the corresponding author.

## Supplementary Materials

The following supporting information can be downloaded at: https://www.mdpi.com/article/10.3390/s24196162/s1, Video S1: Page turning using assistive robot with low-degree-of-freedom hand.

## Appendix A

### Size Parameters and Specifications of Manipulator and Robotic Hand

The size parameters of the manipulator and hand mechanism are defined in Figure A1. Table A1 shows the specifications of the robot.

**Table A1:** Specifications of robot.

Overall length of wheeled platform	450 mm
Overall height of wheeled platform	270 mm
Radius of front wheels	15 mm
Radius of rear wheels	90 mm
Wheelbase	340 mm
Length of upper arm link of manipulator ($l_{uL}$)	300 mm
Length of forearm link of manipulator	
(length from elbow joint to claw mechanism, $l_{fL}$)	570 mm
Thickness of wrist ($t_{W}$)	78 mm
Width of robot base ($b_{B}$)	84 mm
Thickness of robot base ($t_{B}$)	86 mm
Length of U-Link ($l_{U}$)	132 mm
Length of F-Link ($l_{F}$)	104 mm
Thickness of fingers ($t_{F}$)	30 mm
Width of fingers ($b_{F}$)	154 mm
